# Roles of AMPK and Its Downstream Signals in Pain Regulation

**DOI:** 10.3390/life11080836

**Published:** 2021-08-16

**Authors:** Shenglan Wang, Yi Dai

**Affiliations:** 1 School of Acupuncture-Moxibustion and Tuina, Beijing University of Chinese Medicine, Beijing 100029, China; 2 Department of Pharmacy, School of Pharmacy, Hyogo University of Health Sciences, Kobe 650-8530, Japan; 3 Traditional Medicine Research Center, Chinese Medicine Confucius Institute, Hyogo College of Medicine, Kobe 663-8501, Japan; 4 Department of Anatomy and Neuroscience, Hyogo College of Medicine, Nishinomiya 663-8501, Japan

**Keywords:** AMPK, pain, neuron, glia, metabolic sensor

## Abstract

Pain is an unpleasant sensory and emotional state that decreases quality of life. A metabolic sensor, adenosine monophosphate-activated protein kinase (AMPK), which is ubiquitously expressed in mammalian cells, has recently attracted interest as a new target of pain research. Abnormal AMPK expression and function in the peripheral and central nervous systems are associated with various types of pain. AMPK and its downstream kinases participate in the regulation of neuron excitability, neuroinflammation and axonal and myelin regeneration. Numerous AMPK activators have reduced pain behavior in animal models. The current understanding of pain has been deepened by AMPK research, but certain issues, such as the interactions of AMPK at each step of pain regulation, await further investigation. This review examines the roles of AMPK and its downstream kinases in neurons and non-neuronal cells, as well as their contribution to pain regulation.

## 1. Introduction

The International Association for the Study of Pain (IASP) revised the definition of pain in 2020, calling it “an unpleasant sensory and emotional experience associated with, or resembling that associated with, actual or potential tissue damage” [1]. Pain is a protective experience that causes animals to withdraw from or stop harmful stimulation. However, chronic and persistent pain affects quality of life. Pain symptoms that persist or recur for more than 3 months have been defined as chronic pain, which is now regarded as a disease [2]. Chronic pain is a major clinical and public health problem with few effective remedies. Interdisciplinary approaches have recently been applied to pain research. The concept of crosstalk between sensory nervous and metabolic systems has caught the attention among those investigating pain. Emerging evidence indicates that disordered energy homeostasis plays a decisive role in the initiation and development of abnormal sensations, including pain. 

Adenosine monophosphate-activated protein kinase (AMPK) is an evolutionarily conserved serine/threonine enzyme that is ubiquitously expressed in mammalian cells and is described as a cellular fuel gauge [3]. This kinase can be activated by many endogenous stimuli, such as metabolic stress (e.g., heat shock or hypoxia) [3], some hormones associated with lipometabolism and glycometabolism (e.g., adiponectin, leptin) [4,5], pharmacological compounds (e.g., 5-aminoimidazole-4-carboxamide ribonucleotide (AICAR) [6], A769662 [7], resveratrol [8]) and metformin [9]). The activation of AMPK due to the depletion of cellular nutrients or pharmacological intervention prompts catabolic pathways to generate ATP and dampens anabolic pathways to inhibit ATP consumption. Thus, AMPK participates in the regulation of many physiological and pathological processes and is crucial for regulating autophagy, responding to metabolic stress, and maintaining intracellular energy homeostasis. 

As a highly conserved cellular energy sensor, AMPK might play an important role in pain sensation, as it regulates the excitability of ion channels in primary afferent neurons, as well as the activity of spinal microglia [10,11,12]. In addition, AMPK activation participates in pain sensation associated with metabolic disorders, such as diabetes and obesity [10,13]. Moreover, experimental animal and clinical studies have shown that AMPK activators exert analgesic effects [14]. These pieces of evidence, provided herein, support the notion that AMPK and its downstream molecules are key regulators of pathological pain and help us to examine the underlying molecular mechanisms.

## 2. AMPK Subunits and Their Activators in Pain Regulation

**Figure 1:**
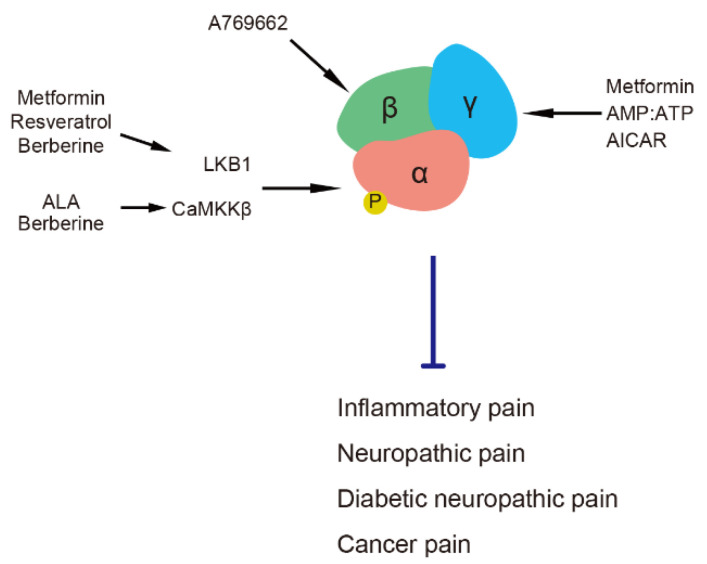
AMPK subunits and their activators in pain regulation. AICAR, 5-aminoimidazole-4-carboxamide ribonucleotide. ALA, α-lipoic acid. AMPK, adenosine monophosphate-activated protein kinase. CaMKKβ, calmodulin-dependent protein kinase kinase β. LKB1, liver kinase B1.

Functional AMPK is an αβγ heterotrimer comprising catalytic α (α1 and α2); scaffolding β (β1 and β2); and regulatory γ1, γ2 and γ3 subunits [15]. An increased intracellular ratio of AMP and ATP allosterically activates AMPK by binding AMP to its γ subunit and causing the phosphorylation of threonine (Thr) 172 in the α subunit [15,16]. Some intracellular kinases, such as liver kinase B1 (LKB1) [17,18,19], calmodulin-dependent protein kinase kinase β (CaMKKβ) [20,21] and transforming growth factor beta-activated kinase-1 [22] target the α subunit to phosphorylate the Thr 172 residue. The phosphorylation of Thr 172 at the α subunit results in 1,000-fold activation of AMPK activity [23]. Activation of the AMPK β subunit also participates in AMPK activity by inducing an allosteric change in kinase that protects AMPK from Thr 172 dephosphorylation [24]. Pain is regulated by AMPK via the modulation of its various subunits by different activators (Figure 1). AICAR, a specific and cell-permeable precursor of 5-amino-4-imidazol-ecarboxamide ribotide (ZMP), mimics regulation of the AMPK γ subunit by AMP to allosterically activate AMPK [6]. Metformin (a prevalent antidiabetic drug) and resveratrol (a natural phenol) activate AMPK through its upstream kinase, LKB1, to phosphorylate the AMPK α subunit [25,26]. The role of the AMPK γ1 subunit is more important in AMPK activation by metformin, the deletion of which determines the phosphorylation of Thr 172, albeit with intact α and β subunits [27]. The natural antioxidant, α-lipoic acid (ALA), activates AMPK via CaMKKβ [28]. Berberine, a natural isoquinoline alkaloid, activates AMPK via either LKB1 or CaMKKβ [29]. The above activators have been applied as analgesics in experimental models of inflammatory pain, nerve injury, painful diabetic neuropathy and cancer pain [10,30,31,32,33,34,35,36,37,38,39]. Moreover, the short-term administration of metformin or AICAR can alleviate diabetic neuropathic pain without anti-diabetic effect [10,40]. Metformin and ALA used in clinic also affect the symptoms of several types of pain [41,42,43,44]. The AMPK α1 subunit plays a specific role in axon regeneration after nerve injury but not the α2 subunit [45]. A specific AMPK β subunit activator, A769662 [7], decreases neuronal hyperexcitability induced by pain-promoting endogenous mediators, such as nerve growth factor (NGF) in dorsal root ganglion (DRG) and trigeminal ganglion neurons [38]. A769662 also acts as a direct blocker/modulator of voltage-gated sodium channels (Na_v_s), such as Na_v_1.7, and ameliorates pain behavior in animal models [46].

## 3. Downstream Signals of AMPK in Pain Regulation

Protein synthesis and metabolism are regulated by AMPK and its downstream kinases through the regulation of protein translation by its downstream mammalian target of rapamycin complex 1 (mTORC1). The mTORC1 pathway is activated in animal models of neuropathy, including chronic constriction injury (CCI) [47,48], spinal nerve ligation (SNL) [38] and spared nerve injury (SNI) [38], as well as the animal models of inflammatory pain [49,50,51]. The inhibition of the mTORC1 pathway by AMPK activators leads to pain relief [38,52,53]. The three core components of mTORC1 are mTOR, a regulatory protein associated with mTOR (raptor) [54] and mammalian lethal with Sec13 protein 8 (mLST8, also known as GßL) [55], and they function as a signaling hub in coordinating cell growth and metabolism [56,57]. Raptor functions as a scaffold to recruit mTOR substrates to mTORC1 and is involved in the phosphorylation of its downstream kinases such as eukaryotic initiation factor-4E-binding protein (4EBP) [58] and p70 ribosomal S6 kinase (S6K) [59]) to promote mRNA translation, and negatively regulate mTORC1 through phosphorylation at serine (Ser) 722 or 792 to bind 14-3-3 protein [60]. Through the direct phosphorylation of raptor, AMPK negatively regulates mTORC1 to interfere with the formation of raptor/mTOR complexes [60] or by inhibiting the mTOR negative regulator tuberous sclerosis complex 2 (TSC2) [61]. Messenger RNA possesses a 5′ cap and a 3′ poly (A) tail. The phosphorylation of the mTORC1 pathway induces the release of eukaryotic translation initiation factor (elF4E), which binds to the mRNA cap and initiates translation [62]. The mTORC1/elF4E pathway is enhanced in models of chronic pain [63,64,65] and initiates cap-dependent translation to rapidly induce protein synthesis in response to pro-inflammatory factors. These processes contribute to mechanical and thermal hyperalgesia [63,64]. The Ser 209 residue on elF4E is a key phosphorylation site for the regulation of cap-dependent translation. Deficient Ser 209 phosphorylation on elF4E attenuates NGF and interleukin-6 (IL-6)-induced neuron excitability, as well as concomitant hypersensitive behavior in mice [63,64]. In addition to participating in pain generation, cap-dependent translation itself can maintain mTORC1, which is essential for the persistence of pain [65]. Mammalian TORC1 and its downstream molecules are found in 35–42% of myelinated fiber marker (N52)-positive neurons, but only 3–5% in CGRP-positive fibers [66]. The inhibitors of mTORC1 increase the thermal threshold of A-fiber but not C-fibers [66,67,68], the paw withdrawal threshold in animal models of neuropathic pain and in healthy animals suggesting that the mTORC1 pathway in primary sensory neurons contribute to basal sensory function [66,68].

**Figure 2:**
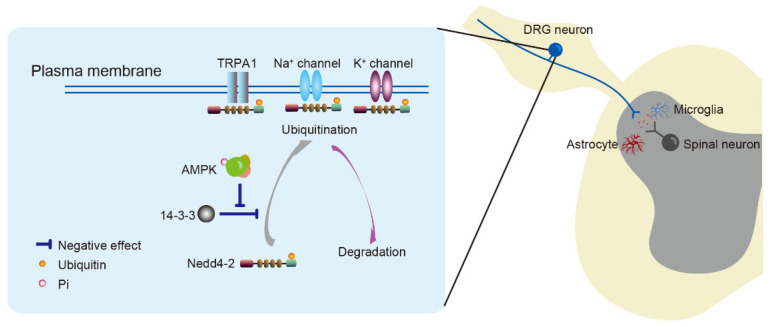
Neuronal excitability is regulated by the AMPK/Nedd4-2 pathway. AMPK activation inhibits the binding of 14-3-3 with Nedd4-2 to increase Nedd4-2-mediated ubiquitination, which results in ion channel (e.g., TRPA1, Na_v_1.7, Na_v_1.8, Kv2.1) degradation in nociceptors. These events can subsequently suppress neuronal excitability. AMPK; adenosine monophosphate-activated protein kinase. DRG; dorsal root ganglion. Nedd4-2; neural precursor cell expressed developmentally downregulated 4-like kinase.

The downstream kinase, neural precursor cell expressed developmentally downregulated 4-like (Nedd4-2), is involved in pain regulation by AMPK via posttranslational modification [69]. Protein ubiquitination is caused by Nedd4-2 catalyzing the transfer of ubiquitin from the E2 ubiquitin-conjugating enzyme to substrate proteins [70]. AMPK promotes the interaction of Nedd4-2 with substrates by inhibiting Nedd4-2 binding to 14-3-3 proteins [71,72] (Figure 2). The role of Nedd4-2 has been determined by the specificity of its bound substrate protein. Sensory neurons express Nedd4-2 that interacts with ion channels, such as potassium channels, sodium channels and transient receptor potential (TRP) channels, as well as transporters such as dopamine transporter and glutamate transporter, and participates in their intracellular trafficking and degradation [40,70,73,74]. These ion channels and transporters in primary sensory neurons participate in maintaining resting potentials and regulating action potentials for neuronal function under physiological and pathological conditions. Nedd4-2 maintains ion channel expression in the plasma membrane and neuronal steady state. The expression of Nedd4-2 is abnormal in the sensory neurons of animal models of neuropathy [40,73,74]. Models of nerve injury have fewer Nedd4-2-positive DRG neurons [73,74], and the plasma membrane expression of Nedd4-2 is decreased in mice with type 2 diabetic neuropathy [40]. The abnormal expression or function of Nedd4-2 results in ion channels’ accumulation in the plasma membrane and subsequent neuronal depolarization.

**Figure 3:**
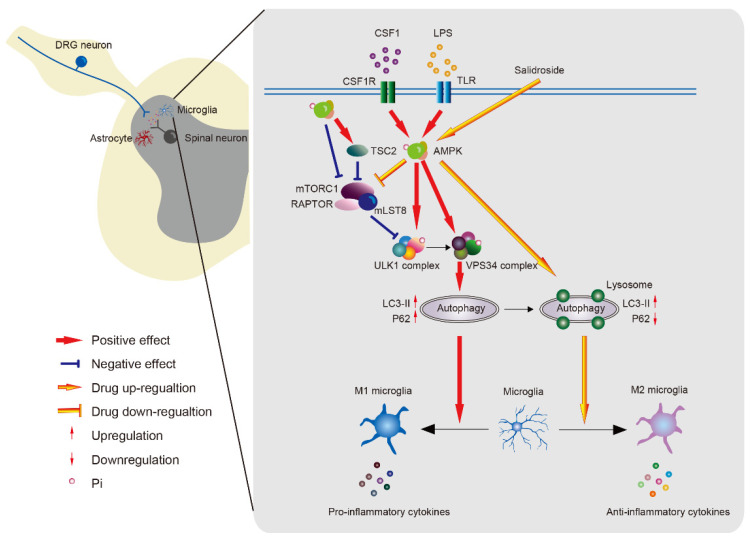
The role of AMPK on neuroinflammation in microglial cells. AMPK induces autophagy by the direct phosphorylation of ULK1 or VPS34 and the indirect activation of ULK1 via inhibiting the mTORC1 pathway. Microglia can be stimulated to an M1 phenotype to release proinflammatory factors by defective autophagy induced by AMPK in pathological condition, while AMPK activator promotes microglial polarization to the M2 phenotype to release anti-inflammatory factors. AMPK; adenosine monophosphate-activated protein kinase. CSF1; colony stimulating factor 1. CSF1R; colony stimulating factor 1 receptor. DRG; dorsal root ganglion. LC3-II; microtubule-associated protein 1A/1B-light chain 3 (autophagosome membranes-associated form). LPS, lipopolysaccharides. mLST8, mammalian lethal with Sec13 protein 8. mTORC1, mammalian target of rapamycin complex 1. RAPTOR, regulatory protein associated with mTOR. TSC2, tuberous sclerosis complex 2. ULK1, unc-51 like autophagy activating kinase 1. VPS34, vacuolar protein sorting 34.

AMPK is a upstream kinase of unc-51 like autophagy activating kinase 1 (ULK1), vacuolar protein sorting 34 (VPS34) or beclin-1, which are key regulators of autophagy initiation and progression [75]. Autophagy is an intracellular process that determines the destination of a protein and its function [76,77]. The phosphorylation of ULK1 promotes the formation of autophagy-specific and VPS34 complexes to produce the autophagosome membrane [78]. During this process, microtubule-associated protein 1A/1B-light chain 3 (LC3) is transformed from the LC3-I (cytosolic form) to the LC3-II (autophagosome membranes-associated form) [79,80]. Increased LC3-II and decreased p62, a substrate of autophagy, indicate the normal autophagic flux that induces protein degradation and damaged organelle removal [81] (Figure 3). In the SNL model, beclin-1, which is a key factor in the VPS34 complex, and LC3, in the ipsilateral spinal dorsal horn, are upregulated on day 7 and maintained for 14 days, suggesting that nerve injury triggers autophagy [82,83]. Increased LC3-II and p62 levels have indicated that defective autophagy is associated with neuropathic pain in a model of nerve injury [83]. Endogenous algesic substances, such as colony stimulating factor 1 (CSF1) or lipopolysaccharides (LPS), increase ULK1 phosphorylation and LC3-II expression to induce autophagy and promote the release of pro-inflammatory factors in vitro [11,12]. AMPK directly phosphorylates ULK1 at Ser 317, Ser 555 and Ser 777 [84,85,86] and indirectly activates it by negative regulating the mTORC1 pathway [87]. Both VPS34 and beclin-1 are also phosphorylated by AMPK to enhance autophagic activity [88]. Activator of AMPK can prevent defective autophagy flux to inhibit pro-inflammatory factors [12]. Collectively, AMPK might regulate pain through the control of various kinases during autophagy.

The peroxisome proliferator-activated receptor γ coactivator-1α (PGC-1α) and acetyl-CoA carboxylase 1 (ACC1) are two important downstream kinases of AMPK in the regulation of mitochondrial biogenesis and cellular energy metabolism to provide neuroprotection [89,90,91]. AMPK directly phosphorylates PGC-1α at Thr 177 and Ser 538 and increases PGC-1α-regulated transcription to promote mitochondrial biogenesis [92], while phosphorylates ACC1 at Ser 79 promote fatty acid oxidation and inhibit fatty acid synthesis [93]. Although lacking in direct evidence of PGC-1α and ACC1 on pain regulation, both of them are involved in oxidative-stress-induced response [94,95], which has a link to pain induction or modulation [96].

## 4. Potential Mechanism of Neuronal AMPK in Pain Regulation

### 4.1. Modulation of Neuronal Excitability in Nociceptors by AMPK

TRP channels comprise a family of non-selective cation channels permeable to Ca^2+^, Na^+^ and K^+^ [97]. Several TRP channels, such as TRPV1 and TRPA1, are expressed in nociceptors to transduce noxious stimuli [98]. The TRPA1 channel responds to noxious stimuli, such as chemical, thermal or mechanical stimuli, to depolarize the resting membrane potential of neurons and can be sensitized in experimental animal pain models [99,100]. The activation of TRPA1 is negatively regulated by AMPK within minutes in rat DRG neurons [10,40]. The activity of AMPK is impaired during the early stage of hyperglycemia in *db*/*db* mouse models of type 2 diabetes and is associated with a concomitant increase in membrane TRPA1 and mechanical allodynia. Metformin suppresses diabetic mechanical allodynia through AMPK activity [10]. Since TRPA1 is ubiquitinated through the Nedd4-2 pathway, abnormal Nedd4-2 distribution causes TRPA1 membrane accumulation in DRG neurons and produces mechanical allodynia in *db*/*db* mice with type 2 diabetes. As an upstream kinase of Nedd4-2, AMPK activation restores membrane-associated TRPA1 and suppresses diabetic mechanical allodynia [40] (Figure 2). Consistent with these findings, total and phosphorylated AMPK is downregulated and accompanied by increased TRPA1 in DRG neurons in an animal model of osteoarthritis, while metformin suppressed osteoarthritic pain by activating AMPK to inhibit TRPA1 [101].

The activity of AMPK participates in the regulation of action potentials in sensory neurons [38]. Many Na_v_s and voltage-gated potassium ion channels in sensory neurons are regulated by Nedd4-2-mediated ubiquitination and degradation. Among them, the potassium ion channels KCNQ2, KCNQ3 and KCNQ5 are expressed in DRG neurons and participate in nociceptive signal transduction in diabetic neuropathic pain [102]. Most Na_v_s are ubiquitinated by interacting with Nedd4-2 via their PY motif [103]. Na_v_1.1-1.9 (but not Na_v_1.4) has been detected in DRG neurons [104]. The downregulation of Nedd4-2 in DRG neurons after nerve injury upregulates Na_v_1.3, Na_v_1.7 and Na_v_1.8, which might be a mechanism of the neuropathic pain [73,74,105,106]. Direct channel phosphorylation by AMPK also regulates ion channel activity [107]. Large conductance calcium- and voltage-activated potassium channel [108], a subunit of the ATP-sensitive potassium channel, inward rectifier potassium ion channel 6.2 (Kir6.2) [109,110], tandem of pore domains in a weakly inward rectifying K+ channel (TWIK)-1-related K+-channel (TREK1), TREK2 [111] and potassium voltage-gated channel (Kv2.1) [112]), which are expressed in sensory neurons [113,114,115,116,117,118,119,120], could be the targets of AMPK phosphorylation. Painful or painless neuropathy or both can develop in patients with diabetes [121,122,123], and the generative mechanism(s) is complicated and is associated with multiple factors, such as the duration of diabetes and age [124,125]. Animal models usually develop hypersensitivity during the early stage of diabetes [10,126], whereas sensation is usually lost after long-term hyperglycemia [127]. Neuronal AMPK can be impaired from early stage of diabetes in mice [10]. Activating AMPK early during the course of diabetes can inhibit neuronal excitability and ameliorate hypersensitivity by downregulating membrane-associated TRPA1 in sensory neurons [10,40]. In contrast, AMPK activation can also enhance neuronal excitability and ameliorate hyposensitivity, including mechanical or thermal responses, by inhibiting the Kv2.1 channel in animal models of long-term diabetic neuropathy [127], suggesting a dual beneficial effect of AMPK activators such as metformin in patients with diabetes.

### 4.2. Axonal AMPK in Nerve Regeneration

Axon damage can cause abnormal neuronal function and even interrupt axonal conduction, resulting in neuropathic pain [128]. Peripheral nerve injury causes an increase in the transcription and translation of mRNA to induce a regeneration response [129,130,131]. The activity of AMPK plays a pivotal role in nerve injury and regeneration in vivo and in vitro.

**Figure 4:**
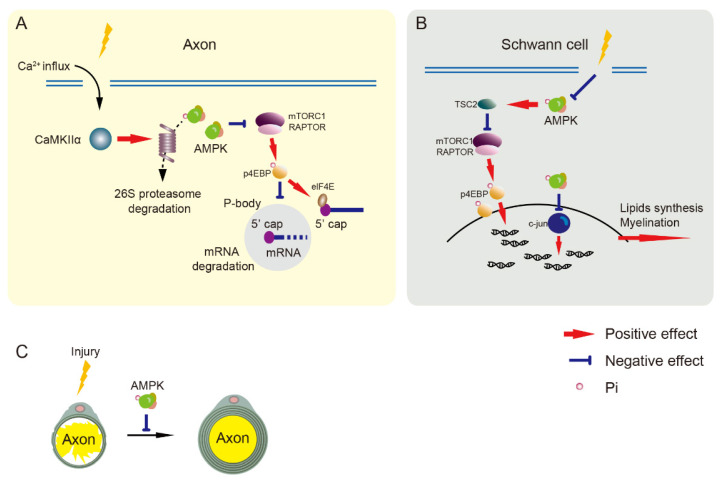
Nerve regeneration is negatively regulated by AMPK. (**A**) The mTORC1 pathway inhibits cap-dependent mRNA degradation to increase transcription. Injury induces calcium influx to activate CaMKIIα/PSMC5 (26S proteasome subunit) pathway and degrades AMPK, resulting in mTORC1 pathway activation in axons. Decreased AMPK activation reduces cap-dependent mRNA degradation that is involved in axon regeneration. (**B**) Nerve injury impairs AMPK activation resulting in the upregulation of mTORC1 and c-jun pathways to enhance translation in Schwann cells. (**C**) AMPK activation inhibits axonal regeneration and Schwann cells-mediated re-myelination. AMPK, adenosine monophosphate-activated protein kinase. CaMKIIα, Ca^2+^/calmodulin-dependent protein kinase II α. elF4E, eukaryotic translation initiation factor. mTORC1, mammalian target of rapamycin complex 1. p4EBP, phosphorylated eukaryotic initiation factor-4E-binding protein. RAPTOR, regulatory protein associated with mTOR. TSC2, tuberous sclerosis complex 2.

Nerve regeneration promotes nerve repair but increases the excitability of nociceptors that result in the maintenance of neuropathic pain [132,133]. Axonal regeneration requires the initiation of transcription and protein synthesis [45,134]. Gene expression is altered by increasing mRNA transport to distal sites of sensory neurons and mRNA translation for nerve regeneration after injury [129,130,131]. Processing bodies (P-bodies) are RNA granules containing RNA decapping enzymes that repress translation and decay mRNA [135], and they can be regulated by mRNA translation signals in sensory neurons [136,137]. Reduced P-bodies in SNI mice, which can be increased by AMPK activators, result in the inhibition of cap-dependent mRNA in sensory neurons and the attenuation of neuropathic pain [136,138]. The central axons of peripheral neurons often do not regenerate after central nerve damage (such as spinal cord injury), whereas axons regenerate in damaged peripheral nerves through the ability of peripheral neurons to self-repair [139]. The signaling pathways involved in transcription and translation change in peripheral, but not central, axons under sciatic nerve injury. This pathway is specifically associated with the AMPKα1 but not the AMPK α2 subunit. Activated AMPKα1 commonly inhibits neuronal axon regeneration. After sciatic nerve axotomy, AMPK expression and activity are gradually reduced in the peripheral axons of DRG neurons. This change is regulated by the phosphorylation of Ca^2+^/calmodulin-dependent protein kinase II α (CaMKIIα) and its downstream proteasome 26S subunit, ATPase 5 (PSMC5). Upregulated PSMC5 and CaMKIIα phosphorylation after sciatic nerve axotomy causes a reduction in AMPKα1 [45] (Figure 4A).

### 4.3. AMPK in Spinal Neurons

The spinal cord dorsal horn is the first site of synapse processing in the pain pathway from the periphery to the brain [140]. Many types of neurons in the spinal cord dorsal horn participate in pain signal integration [141,142,143]. Local oxidative stress after peripheral nerve or spinal cord injury can affect spinal cord neurons. Little is known about the function of spinal AMPK in vivo. Two studies of spinal neurons in vitro find that AMPK might prevent spinal neuron damage by anti-oxidation [144,145]. Activation of the AMPK/PGC-1α pathway participates in the regulation of mitochondrial dysfunction under oxygen and glucose deprivation-induced oxidative stress in spinal neurons [145]. In addition, AMPK activity improves lipid peroxidation and DNA damage and protects spinal neurons from death and apoptosis induced by reactive oxygen species in spinal neurons [144].

## 5. Potential Mechanism of Non-Neuronal AMPK in Pain Regulation

### 5.1. AMPK in Schwann Cells

Because Schwann cells are involved in myelination and are crucial for nerve fiber function, their regulation is essential for nerve regeneration. Schwann cells are derived from neural crest cells that migrate into peripheral nerves and convert to Schwann cell precursors and immature Schwann cells during embryonic development. Immature Schwann cells differentiate into myelinated or non-myelinated phenotypes after birth [146,147]. The sciatic nerves of embryonic rats contain phosphorylated and non-phosphorylated AMPK, but levels gradually decrease from birth to adulthood. Activated AMPK can suppress myelination in newborn rats, suggesting that it negatively regulates developmental myelination and re-myelination after injury [148]. Energy consumption appears to control Schwann cell-mediated myelination during postnatal development. Re-myelination after nerve injury is energy-consuming, and AMPK activity in Schwann cells decreases over time after sciatic nerve injury [148]. AMPK activates c-Jun, which downregulates myelin gene expression, thus negatively regulating myelination, whereas inhibiting mTORC1 suppressed myelin sheath thickness (Figure 4B,C).

### 5.2. AMPK in Macrophages

Activated macrophages can release pro-inflammatory factors that contribute to pain [32,149]. The activity of AMPK plays a comprehensive role in neuroinflammation. AMPK α1 is the dominant subunit in macrophages involved in the regulation of inflammatory pain. The AMPK activators, AICAR [32], metformin [150] and A769662 [149]) that target multiple AMPK subunits suppress the production of microphage-related cytokines, IL-1β, IL-6, as well as NO in animal models of inflammatory pain [32,149]. Moreover, AMPK activators inhibit complete Freund’s adjuvant (CFA)- and LPS-induced nuclear factor kappa B and mitogen-activated protein kinase signaling pathways in macrophages in inflamed local tissues [32,149]. Whereas CFA increases AMPK activation in macrophages, AICAR suppresses CFA-induced pain [32], suggesting that AMPK activation in macrophages is a self-serving process in inflammatory pain. 

### 5.3. AMPK in Glial Cells

Neuroinflammation regulated by microglia and astrocytes is crucial in neuropathic pain [151,152]. The activation of AMPK inhibits the activation of microglial and astrocytes and reduces pro-inflammatory factors, including IL-1β and TNFα, resulting in the amelioration of mechanical allodynia in a rat model of trigeminal neuralgia [153]. After peripheral nerve injury, increased endogenous LPS or CSF1 activates spinal cord microglia [154,155]. Both LPS and CSF1 can activate AMPK, resulting in microglial autophagy [11,12] (Figure 3). The activation of AMPK induced by CSF1 triggers autophagy and promotes the transcription of pro-inflammatory cytokines, which can be inhibited by an AMPK inhibitor or by genetic AMPK knockdown [11]. Another possible mechanism of the analgesic effects of AMPK activators determined from a study of LPS-induced pain is as follows. Since increased p62 and LC3 expression can inhibit autophagy flux, LPS causes microglia to switch on the M1 polarization type and release pro-inflammatory cytokines [12]. The AMPK activator, salidroside, can prevent the subsequent effects of LPS and activates autophagy flux to bind to lysosome, which increases the M2 microglia to release anti-inflammatory factors [12]. Although AMPK regulates several downstream targets, the mechanism of AMPK effects in non-neuronal cells seems to be selective, and the mTORC1 pathway is not involved in the regulation of spinal non-neuronal cells in a model of neuropathic pain [48]. Controversially, exogenous LPS decreases AMPK activity in microglial cells [156]. AMPK activity is not increased in a model of CCI [157]. Why AMPK activity differentially changes in these in vitro and in vivo experiments and through which mechanism remain unclear; nevertheless, that AMPK plays a crucial role in the regulation of pro-inflammatory factors in both inflammatory and neuropathic pain is not in any doubt.

### 5.4. AMPK in Blood Mononuclear Cells (BMCs)

In addition to cells in injured tissues, AMPK regulates pain through its function in BMCs. The nucleotide-binding domain (NOD)-like receptor family, pyrin domain containing 3 (NLRP3) inflammasome complex in BMCs is a critical component that activates innate immune defenses through the mediation of caspase-1 activation and the secretion of proinflammatory IL-1β and IL-18 [158]. AMPK can inhibit NLRP3 inflammasome complex formation by regulating autophagy [159,160], PGC-1α involvement in mitochondrial function [161] and endoplasmic reticulum stress [162,163]. Inhibiting AMPK activation induces hyperalgesia associated with NLRP3 inflammasome protein activation and increased serum levels of IL-1β and IL-18. Notably, AMPK activation is deficient and the NLRP3 inflammasome axis is overactivated in blood cells from patients with fibromyalgia, which is a prevalent chronic pain disease. Daily metformin intake can improve the biochemical index in blood cells, as well as clinical symptoms of pain, depression and tender areas of pain, by increasing AMPK activation in patients with fibromyalgia [43].

## 6. Conclusions

Multiple types of cells in mammalian peripheral and central nervous systems ubiquitously express AMPK. Accumulating evidence indicates that AMPK plays important roles in pain modulation. Considering its multiple intracellular downstream signaling pathways, AMPK might serve as an intersection of the mechanism(s) of pathological pain. The diversity of AMPK activity among cell types and pathological states might reflect the complexity of pain pathology. Therapeutic targets of AMPK might simultaneously resolve the problems associated with metabolic disorders and pain. Further clinical trials of AMPK activators will benefit patients with painful disease states.

## Data Availability

Not applicable.
